# Supplementary material for: Postprandial sleep mechanics in Drosophila
Source: eLife. 2016 Nov 22;5:e19334. doi: 10.7554/eLife.19334 (PMC5119887; doi:10.7554/eLife.19334)
Supplement: Supplementary file 1. — Flowchart depicting the algorithm used for processing dye-reference mark pixel distance data to identify feeding events. DOI: http://dx.doi.org/10.7554/eLife.19334.020 [file elife-19334-supp1.docx]

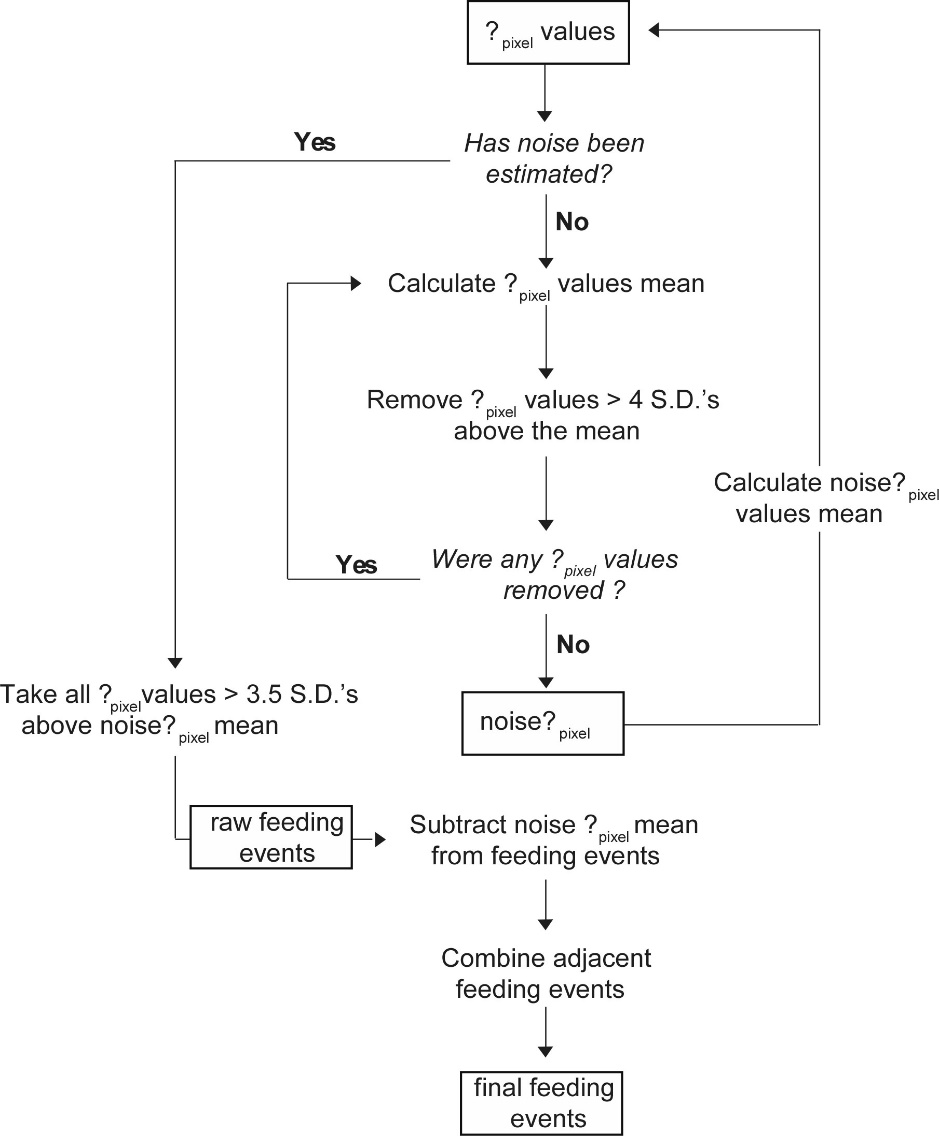


**Supplementary File 1. Meal selection algorithm**

Flowchart depicting the algorithm used for processing dye-reference mark pixel distance data to identify feeding events.
